# Non-occupational exposure to paint fumes during pregnancy and risk of congenital anomalies: a cohort study

**DOI:** 10.1186/1476-069X-11-54

**Published:** 2012-08-14

**Authors:** Dorrit Hjortebjerg, Anne-Marie Nybo Andersen, Ester Garne, Ole Raaschou-Nielsen, Mette Sørensen

**Affiliations:** 1 Danish Cancer Society Research Centre, Copenhagen, Denmark; 2 Section of Social Medicine, Department of Public Health, University of Copenhagen, Copenhagen, Denmark; 3 Pediatric Department, Hospital Lillebaelt, Kolding, Denmark

**Keywords:** Epidemiology, Organic solvent, Paint fumes, Birth cohort, Congenital anomalies, Birth defects

## Abstract

**Background:**

Occupational exposure to organic solvents during the 1^st^ trimester of pregnancy has been associated with congenital anomalies. Organic solvents are also used in the home environments in paint products, but no study has investigated the effect of such exposure in a general population.

**Methods:**

We studied associations between residential exposure to paint fumes during the 1^st^ trimester of pregnancy and predefined subgroups of congenital anomalies, using data from the Danish National Birth Cohort (DNBC). During 2001 and 2003, a total of 20 103 pregnant women, enrolled in the DNBC, were interviewed in the 30^th^ week of gestation about the use of paint in their residence during pregnancy. By the end of first trimester, information about smoking habits, alcohol consumption and occupation were collected. Information on congenital anomalies was obtained from national registers. Associations were examined by estimating odds ratios (OR) using logistic regression.

**Results:**

In total 1404 women (7%) had been exposed to paint fumes during the 1^st^ trimester of pregnancy and 1086 children were diagnosed with congenital anomalies; 73 children with congenital anomalies had been exposed to paint fumes in utero. Exposure to paint fumes seemed positively associated with congenital anomalies of the nervous system (OR 2.19, 95% confidence interval (CI) 0.76 to 6.32), ear, face and neck (OR 2.15, 95% CI 0.84 to 5.55) and the renal system (OR 2.16, 95% CI 1.02 to 4.58) after adjustment for maternal age, smoking, alcohol consumption and occupational solvent exposure. Congenital anomalies in the remaining subgroups were not associated with the exposure.

**Conclusions:**

Our results suggest that in the general population, exposure to paint fumes during the 1^st^ trimester of pregnancy may increase the risk of some types of congenital anomalies, but the findings need to be confirmed.

## Introduction

The prevalence of congenital anomalies has been estimated to 50 per 1000 in live births in Denmark. Congenital anomalies are associated with significant societal costs related to treatment and improving quality of life with medical, social and educational services. Furthermore, congenital anomalies are of the top 20 list of leading causes of burden of disease (DALY´s) and are an important contributor to infant mortality. Also, the World Health Organisation estimated in 2004 that 260 000 deaths or about 7% of all neonatal deaths were attributable to congenital anomalies.

For a majority of the congenital anomalies, the etiology is unknown, though occupational and environmental agents are suspected to be involved [1]. Organic solvents are widely used in the work environment, e.g. the graphic industry and dry-cleaning, and in the home environment in products such as paint and cleaning agents. They represent a structurally diverse group of chemicals with low molecular weight, lipophilicity and are able to dissolve other organic substances. Chemicals in the solvent class include aliphatic hydrocarbons, aromatic hydrocarbons, halogenated hydrocarbons, aliphatic alcohols, glycols and glycol ethers. They are volatile liquids at room temperature and their main routes of exposure are through inhalation and skin contact.

Epidemiological studies have indicated that women occupationally exposed to organic solvents during pregnancy may have a higher risk for congenital anomalies [2-7] than unexposed women, though the results are far from consistent [3,5,8]. Some studies have indicated that specific organic solvents like halogenated hydrocarbons, e.g. per- and trichloroethylene [9] and aromatic hydrocarbons, e.g. toluene and xylene [3] might be more hazardous to the fetus than other organic solvents.

Ethanol is the most known and widely used organic solvent. Excessive intake of ethanol during pregnancy may lead to the foetal alcohol syndrome. This syndrome includes characteristic facial anomalies of the affected child, growth retardation and permanent central nervous system damage and is also associated with an increased risk of congenital anomalies in several organ systems. A suggested mechanism of action is that ethanol and the degradation product acetaldehyde may induce cell death and abnormal cell migration, which can cause a variety of congenital anomalies [10]. A similar mechanism of action, have been suggested for exposure to the organic solvents toluene or gasoline and risk of congenital anomalies [11,12].

To our knowledge, no studies have investigated if exposure to organic solvents in the home environment during pregnancy affects the risk for congenital anomalies in a general population. Organic solvents are used in different concentrations in all kinds of paint and many of these are liberated during painting and subsequently during drying and hardening. Many pregnant women are expected to be exposed to organic solvents from paint fumes during pregnancy due to “nesting behavior” and/or moving residence.

The aim of the present study was to investigate the association between exposure to paint fumes in the residence during the 1^st^ trimester of pregnancy and the risk of congenital anomalies in a prospective cohort.

## Methods

### Study population

The study was carried out within The Danish National Birth Cohort (DNBC), which is a population based cohort of more than 100 000 pregnant women and their offspring, and created to study determinants of early child health and diseases in later life [13]. From March 1996 to November 2002 pregnant women, who met the requirements of being able to speak Danish, being pregnant and intended to carry the pregnancy to term, were invited to participate in the DNBC. The invitation took place at the general practitioner, at the first antenatal visit, where the women received written information and an informed consent to sign and forward to the study secretariat. In the DNBC the women took part in two prenatal computer-assisted telephone interviews around gestational week 12 and 30. The content of the interviews was developed in consultation with external experts and included, among others, questions related to lifestyle factors such as alcohol consumption and smoking habits and furthermore questions about occupation. The Danish ethical committee approved the DNBC.

### Paint fumes exposure assessment

In the time period between September 2001 and May 2003 the second prenatal interview included questions about exposure to paint fumes in the residence. In total 20 103 pregnant women were interviewed during this time period. At first the women were asked if any painting had been done in their residence during pregnancy and if so, if they painted “furniture, floor, radiator and/or woodwork” and/or “wall and/or ceiling” and further, in exactly which gestational week(s) these two categories of painting was done.

From these questions, we generated the variable “exposure to paint fumes in 1^st^ trimester” (no/yes) based on painting done in 1-12^th^ pregnancy week, regardless of the object of painting (furniture, floor, radiator and/or woodwork, wall and/or ceiling).

### Assessment of congenital anomalies

Information on congenital anomalies in the offspring were obtained by linking the unique personal identification number of the mother and her child to the nationwide National Hospital Discharge Registry, which entails information on all hospital admissions and outpatient contacts on the individual patient [14]. All pregnancy outcomes are reported to and recorded in this register including congenital anomalies in live born children, whereas congenital anomalies in stillbirths, abortions and terminated pregnancies are not registered in the National Hospital Register. Congenital anomalies in live born children are diagnosed and assigned by physicians according to the International Classification of Diseases 10^th^ Revision (ICD-10). We identified all children with ICD-codes Q00.0 to Q99.9 recorded during the first three and a half year of life.

Congenital anomalies were grouped according to the EUROCAT recommendations [15], in specific subgroups, mainly based on organ systems and in addition genetic syndromes and other anomalies were considered as one group. EUROCAT, which is a network of population-based registries for the epidemiologic surveillance of congenital anomalies, recommend in their guideline to exclude both minor isolated anomalies and non-congenital anomalies. Since minor anomalies which include defects of smaller medical, functional or cosmetic importance, may be associated with exposure to chemical substances, such as organic solvents, we did not choose to exclude minor isolated anomalies, as purposed by EUROCAT. However, the following ICD-codes were excluded from our study: Q40.0 (pyloric stenosis), Q67.3 (plagiocephaly – head asymmetry), Q68.0 (torticollis) and Q75.3 (macrocephalus) were all excluded because they mainly occur at birth or after birth, and thus, unlikely to be associated with paint fumes exposure during the first trimester of pregnancy; Q38.1 (tongue tie – short frenum) and Q27.0 (hypoplasia of umbilical artery) were excluded because these are often not considered as congenital anomalies; Q40.1 (hiatus hernia) was excluded since it most often does not cause any symptoms and/or often disappears within the first years of life; Q32.0 (tracheomalacia) and Q31.4-Q31.5 (laryngomalacia) representing weakness and floppiness of the walls of the trachea were excluded since these conditions most often disappear within 18 month of age. Furthermore, infants and children with the ICD-codes; Q90.0-Q99.9, which included chromosomal abnormalities, were not considered as events.

The congenital anomaly subgroups used were: nervous system (Q00-Q07), eye (Q10-Q15), ear, face and neck (Q16-Q18), congenital heart defects (Q20-Q28), respiratory system (Q30-Q34), oro-facial clefts (Q35-Q37), digestive system (Q38-Q45, Q790), abdominal wall defects (Q792, Q793), renal (Q60-Q64), genital (Q50-Q56), limb defects (Q650-Q669, Q680-Q74), musculo-skeletal (Q670-Q678, Q75-Q789) and other congenital anomalies (Q80-Q85, Q87, Q89). Distribution of the specific congenital anomalies according to exposure status among the 1086 cases in the study using ICD10 is shown in Additional file 1.

### Covariates

We decided a priori to adjust for the following potential confounders: maternal smoking (no, yes), alcohol consumption (< 1, ≥ 1 drinks per week), potential occupational exposure to organic solvents and maternal age.

Information on smoking habits, alcohol consumption and occupation came from the first (12^th^ week) interview. An industrial hygienist defined “potentially occupationally exposed to organic solvents” as house painters, dry cleaners, employers in graphic industries, lab technicians and hairdressers. Maternal age was obtained from the National Hospital Discharge Registry.

### Statistical analyses

We used logistic regression (proc GENMOD, SAS) and estimated odds ratios (OR) to test for associations between exposure to paint fumes in the 1^st^ trimester of pregnancy (no/yes) and 1) all congenital anomalies and 2) congenital anomalies by subgroup. We performed crude analyses and analyses adjusted for the a priori defined potential confounders. We calculated two-sided 95% confidence intervals (CI) based on Wald’s test. All analyses were done in SAS (version 9.1, SAS Institute, Inc., Cary. NC, USA).

## Results

Of the pregnant women, interviewed between September 2001 and May 2003 and with a singleton outcome (N = 20 103), we excluded women, who gave birth to a stillborn child (N = 57), whose children had a diagnosis of chromosomal abnormalities (N = 33), who had incomplete information on the use of paint during the 1^st^ trimester (N = 55) and with incomplete information on any potential confounder (N = 15). Also, for women participating with two pregnancies/births within the study period, we excluded the second pregnancy/birth (N = 8). The remaining 19 935 mother and child pairs were eligible for the analyses.

Characteristics of the women are shown in Table 1. In total 1404 women (7%) were exposed to paint fumes in their residence in the 1^st^ trimester. Among the 1086 children recorded in the Danish Hospital Discharge Register with congenital anomalies, 73 children had mothers, who had been exposed to paint fumes during the 1^st^ trimester of pregnancy (Table 1).

**Table 1:** Characteristics of the 19935 women and child pairs, from the Danish National Birth Cohort, exposed and not exposed to paint fumes in their residence during 1st trimester of pregnancy

	**Exposed to paint fumes**				**Not exposed to paint fumes**				P*
	**N**	**(%)**	**Mean ± std**		**N**	**(%)**	**Mean ± std**		
All women	1404	(7)			18531	(93)			
Painting of:									
Furniture, floor, radiator and/or woodwork	147	(10)							
Wall and/or ceiling	853	(61)							
Both of the categories above	404	(29)							
Age of the women (years)			29.2	±4.2			29.3	±4.3	0.52
Smoking during 1st trimester									0.21
No	1048	(75)			14107	(76)			
Yes	356	(25)			4424	(24)			
Average alcohol consumption (drinks/week) during 1st trimester									0.52
<1	1073	(76)			14301	(77)			
≥1	331	(24)			4230	(23)			
Working with organic solvents at first (12th week) interview									0.10
No	1350	(96)			17965	(97)			
Yes	54	(4)			566	(3)			
Children diagnosed with congenital malformation									0.62
All	73	(5)			1013	(5)			
One congenital malformation	57	(4)			815	(4)			
More than one congenital malformation	16	(1)			198	(1)			

*t -test (continuous) and *x *^2^ (non-parametric).

There was no increased risk with exposure to paint fumes in the 1^st^ trimester of pregnancy for all congenital anomalies combined (OR 0.95, 95% CI 0.74 to 1.21). Looking at individual subgroups, exposure to paint fumes in the 1^st^ trimester of pregnancy was associated with a more than twofold increased risk of congenital anomalies in the nervous system (OR 2.19, 95% CI 0.76 to 6.32), ear, face and neck (OR 2.15, 95% CI 0.84 to 5.55) and in the renal system (OR 2.16, 95% CI 1.02 to 4.58) (Table 2). The remaining subgroups were not persuasively associated with the exposure. Further adjustment of the analyses by the mother’s occupational status did only result in minor changes in the estimates (results not shown). In the subgroup: genital anomalies, we performed a sub-analysis in which we restricted the analysis to boys only. This only resulted on minor change in the estimate (results not shown).

**Table 2:** Association between exposure to paint fumes in the residence during 1st trimester of pregnancy and congential anomalies among 19935 women and child pairs from the Danish National Birth Cohort

	**Cases**	**Non-cases**	**Crude**	Adjusted*
	**N**	**N**	**OR (95 % CI)**	**OR (95 % CI)**
All congenital malformation				
Not exposed	1013	17518	1.00 (Ref)	1.00 (Ref)
Exposed	73	1331	0.95 (0.74-1.21)	0.95 (0.74-1.21)
Nervous system				
Not exposed	24	18507	1.00 (Ref)	1.00 (Ref)
Exposed	4	1400	2.20 (0.76-6.36)	2.19 (0.76-6.32)
Eye				
Not exposed	37	18494	1.00 (Ref)	1.00 (Ref)
Exposed	5	1399	1.79 (0.70-4.55)	1.79 (0.70-4.57)
Ear, face and neck				
Not exposed	31	18500	1.00 (Ref)	1.00 (Ref)
Exposed	5	1399	2.13 (0.83-5.49)	2.15 (0.84-5.55)
Congenital heart defects				
Not exposed	156	18375	1.00 (Ref)	1.00 (Ref)
Exposed	9	1395	0.76 (0.39-1.49)	0.76 (0.39-1.49)
Respiratory system				
Not exposed	23	18508	1.00 (Ref)	1.00 (Ref)
Exposed	2	1402	0.27 - 4.87	1.13 (0.27-4.79)
Cleft lip and cleft palate				
Not exposed	36	18495	1.00 (Ref)	1.00 (Ref)
Exposed	3	1401	1.10 (0.34-3.58)	1.06 (0.33-3.46)
Digestive system				
Not exposed	44	18487	1.00 (Ref)	1.00 (Ref)
Exposed	2	1402	0.60 (0.15-2.48)	0.61 (0.15-2.50)
Abdominal wall defects				
Not exposed	10	18521	1.00 (Ref)	1.00 (Ref)
Exposed	0	1404		
Renal				
Not exposed	50	18481	1.00 (Ref)	1.00 (Ref)
Exposed	8	1396	2.12 (1.00-4.48)	2.16 (1.02-4.58)
Genital				
Not exposed	220	18311	1.00 (Ref)	1.00 (Ref)
Exposed	14	1390	0.84 (0.49-1.44)	0.83 (0.48-1.43)
Limb defects				
Not exposed	386	18145	1.00 (Ref)	1.00 (Ref)
Exposed	24	1380	0.82 (0.54-1.24)	0.82 (0.54-1.24)
Muscula and skeletal				
Not exposed	45	18486	1.00 (Ref)	1.00 (Ref)
Exposed	6	1398	1.76 (0.75-4.14)	1.77 (0.75-4.16)
Other malformation				
Not exposed	96	18435	1.00 (Ref)	1.00 (Ref)
Exposed	9	1395	1.24 (0.62-2.46)	1.24 (0.62-2.46)

*Adjusted for: Maternal age, smoking during 1st trimester, alcohol consumption during 1st trimester, working with organic solvents at first interview (12th week).

## Discussion

Our results indicate a positive association between exposure to paint fumes in the 1^st^ trimester of pregnancy and the risk of congenital anomalies in the nervous system, the ear, face and neck and the renal system. Some occupational studies have indicated similar results. A case-referent study found that occupational exposure to aromatic solvents during the 1^st^ trimester of pregnancy was associated with congenital anomalies, predominantly in the renal-urinary subgroup and in the gastrointestinal subgroup [2]. Two prospective occupational studies have found associations between maternal exposure to organic solvents and major congenital anomalies. The first study found associations between congenital anomalies in the renal system (hydronephrosis) and in the nervous system, particular neural tube defects [5]. The second study, designed as a cohort study, found occupational exposure to organic solvents to be associated with urinary anomalies, with OR of 2. Furthermore the authors described a weak association with congenital anomalies in the nervous system, in which the observed cases mainly were diagnosed with hydrocephalus [7]. Non-occupational studies have indicated a relation between prenatal alcohol consumption and risk of renal congenital anomalies, but a recent review, which investigated foetal alcohol spectrum disorders and risk of specific anomalies, did not confirm a homogenous pattern according to renal congenital anomalies [16]. Also, some environmental studies have indicated positive associations between solvents in drinking water and neural tube defects, which support our findings regarding congenital anomalies in the nervous system [17,18]. In line with our results there are some indications from earlier occupational and environmental studies of an association between organic solvents and increased risk of congenital anomalies in the nervous system and the renal system [2,5,7]. However, due to small number of cases in these two congenital anomaly groups in the present study (28 and 58, respectively) the results should be treated with caution.

Our study suggests that exposure to paint fumes might increase the risk for congenital anomalies in the ear, face and neck. One prior occupational study failed to find a positive association between exposure to organic solvents and congenital anomalies in the ear, face, and neck. In contrast, these kinds of anomalies have often and predominantly been observed in studies investigating maternal alcohol consumption or solvent abuse during pregnancy [11,12]. Unstable findings due to the small numbers of cases may account for the inconsistent findings.

Some studies have indicated associations between occupational exposure to organic solvents and congenital anomalies in the digestive system or cleft lip and cleft palate [5-7], for which we find no association. This can be due to different groupings of the particular congenital anomaly, but may also be explained by low power in our study to detect associations (only 39 cases) or by exposure to different organic solvents. Organic solvents are a very diverse group of chemicals with different toxicity and may therefore not be expected to create a homogenous pattern.

In our study, estimation of exposure to paint fumes during the 1^st^ trimester of pregnancy is based on questions in the 30^th^ pregnancy week. This involves limitations compared with measurement of concentrations of organic solvents, as many factors affecting the actual concentrations, such as ventilation and room temperature, are not accounted for. Furthermore, paint contains different organic solvents in different concentrations depending on the type and brand of the paint, and, thus, makes it difficult to predict an actual exposure to specific organic solvents when exposure information is based on questions. Also the women had to recall exposure to paint fumes for some months, which could lead to misclassification.

Although the overall ultrasonic examination for structural anomalies and developmental defects as a general offer to all pregnant women were first introduced in Denmark in 2004 (that is after the DNBC enrolment period), it cannot be ruled out, that some pregnant mothers may have been ultrasonic examined on indication resulting in, that they already in the 30^th^ pregnancy week knew, that their child would be born with a congenital anomaly. This can have introduced some recall bias with regard to the questions regarding painting.

We obtained information on congenital anomalies in the National Hospital Register, identifying all children with diagnosis of congenital anomalies from birth until the age of 3.5 year old. The applied time period allowed us to include diagnosis, such as congenital anomalies in the urinary system, which usually not are detected at birth, unless routine ultrasound during pregnancy has been used [7]. The diagnosis may however be subjects to some misclassification, as some anomalies may wrongly be classified as other anomalies. This non-differential misclassification is most likely not associated with the exposure to paint fumes in the residence and would as such, either not affect the risk estimates or bias it towards the neutral value.

Based on EUROCAT´s recommendations, we grouped congenital anomalies in 13 subgroups. For three of these, we found exposure odds ratios higher than two, but only the estimate for the renal subgroup remained statistically significant in the adjusted analysis. Overall the subgroups used are rather large with some etiological heterogeneity. The rationale behind this grouping was that the numbers of congenital anomalies were too small to examine more specific subgroups. However, this might have diluted the effect of more specific groups of anomalies.

In total we found no estimates of exposure ratios to be less than 0.5. We have adjusted for few obvious potential confounding factors and it can be argued that other risk factors should have been included. However, the size of the data material did not allow further adjustment. The fact that the crude and adjusted results were virtually identical may indicate that our results are not largely confounded. On the other hand, information on potential important confounders such as sources of solvent exposure in the home environment including use of cleaning agents or hobbies was not available for the study [19,20]. The consistency between our results and previous findings indicates the associations we find might be true, although it is possible that our findings may be due to chance. The fact that our study is based on small number of exposed cases may have resulted in statistical instability of our findings.

We found congenital anomalies in 5.5% of the pregnancies included in our study and that congenital anomalies were more common in boys than in girls. Both these findings are consistent with Danish national data for live births. Furthermore, our study is based on data from a population based birth cohort, from which we only excluded 1%, mainly because of birth of stillborns and incomplete information on covariates and not the main exposure of interest, paint fumes. However, it is a limitation to the study that we were only able to include live born children with congenital anomalies. Assuming that exposure to paint fumes affects the risk of severe congenital anomalies that may result in abortion or stillbirth, the risk estimates of such anomalies would be underestimated in our study. We also excluded 33 women, whose children were diagnosed with chromosomal abnormalities. These children could not be cases despite other morphological anomalies, since children with chromosomal abnormalities (who often have anomalies) were excluded as potential cases as these conditions are founded before conception and as such, not due to paint fumes exposure during pregnancy. A previous study has shown that participants in the DNBC were somewhat healthier, according to smoking habits than the general population [21], but we have no knowledge of whether the women included in our study have painted more or less than the general population.

## Conclusions

Our results suggested an association between exposure to paint fumes in the 1^st^ trimester of pregnancy and risk of congenital anomalies in the nervous system, the ear, face and neck and the renal system. These results need to be confirmed.

## Abbreviations

DNBC: Danish National Birth Cohort; OR: Odds ratio; CI: Confidence interval; ICD-10: International classification of diseases 10th Revision.

## Competing interests

The authors declare that they have no competing interests.

## Author contributions

DH, MS, ANA and ORN conceived the study. ANA participated in establishing the Danish National Birth Cohort. EG participated in grouping of the congenital anomalies. DH analysed the data. MS, ANA and ORN contributed to the data analysis and data interpretation. DH drafted the paper, and all the authors critically revised it. All authors read and approved the final manuscript.

## Supplementary Material

Additional file 1 **Distribution of congenital anomalies using the International Classification of Diseases 10**^**th **^**Revision (ICD10) among the 1086 cases in the study, according to exposure status.** Click here for file
