# Supplementary material for: Non-occupational exposure to paint fumes during pregnancy and risk of congenital anomalies: a cohort study
Source: Environ Health. 2012 Aug 14;11:54. doi: 10.1186/1476-069X-11-54 (PMC3533823; doi:10.1186/1476-069X-11-54)
Supplement: Additional file 1 — Distribution of congenital anomalies using the International Classification of Diseases 10th Revision (ICD10) among the 1086 cases in the study, according to exposure status. [file 1476-069X-11-54-S1.doc]

**Additional file 1**

Distribution of congenital anomalies using the International Classification of Diseases 10^th^ Revision (ICD10) among the 1086 cases in the study, according to exposure status

| ICD10 code | Exposed to paint fumes during 1st trimester | | Not exposed to paint fumes during 1st trimester | |
| --- | --- | --- | --- | --- |
|  | N | % | N | % |
| All | 102 | 100 | 1320 | 100 |
| Q00 |  |  | 1 | 0.1 |
| Q02 |  |  | 9 | 0.7 |
| Q03 |  |  | 8 | 0.6 |
| Q04 | 3 | 2.9 | 7 | 0.5 |
| Q05 |  |  | 4 | 0.3 |
| Q06 |  |  | 1 | 0.1 |
| Q07 | 2 | 2.0 | 2 | 0.2 |
| Q10 | 4 | 3.9 | 20 | 1.5 |
| Q11 |  |  | 3 | 0.2 |
| Q12 |  |  | 7 | 0.5 |
| Q13 |  |  | 6 | 0.5 |
| Q14 | 1 | 1.0 | 3 | 0.2 |
| Q15 |  |  | 4 | 0.3 |
| Q16 |  |  | 2 | 0.2 |
| Q17 | 1 | 1.0 | 7 | 0.5 |
| Q18 | 4 | 3.9 | 24 | 1.8 |
| Q20 |  |  | 7 | 0.5 |
| Q21 | 6 | 5.9 | 93 | 7.1 |
| Q22 |  |  | 19 | 1.4 |
| Q23 | 1 | 1.0 | 10 | 0.8 |
| Q24 | 1 | 1.0 | 32 | 2.4 |
| Q25 | 4 | 3.9 | 50 | 3.8 |
| Q26 |  |  | 4 | 0.3 |
| Q27 |  |  | 5 | 0.4 |
| Q28 |  |  | 8 | 0.6 |
| Q30 |  |  | 8 | 0.6 |
| Q31 | 2 | 2.0 | 9 | 0.7 |
| Q32 |  |  | 2 | 0.2 |
| Q33 |  |  | 4 | 0.3 |
| Q35 | 1 | 1.0 | 10 | 0.8 |
| Q36 | 1 | 1.0 | 10 | 0.8 |
| Q37 | 3 | 2.9 | 26 | 2.0 |
| Q38 |  |  | 2 | 0.2 |
| Q39 |  |  | 8 | 0.6 |
| Q40 |  |  | 2 | 0.2 |
| Q41 |  |  | 8 | 0.6 |
| Q42 | 1 | 1.0 | 7 | 0.5 |
| Q43 | 1 | 1.0 | 13 | 1.0 |
| Q44 |  |  | 4 | 0.3 |
| Q45 |  |  | 3 | 0.2 |
| Q52 | 1 | 1.0 | 7 | 0.5 |
| Q53 | 11 | 10.8 | 162 | 12.3 |
| Q54 | 2 | 2.0 | 51 | 3.9 |
| Q55 |  |  | 8 | 0.6 |
| Q56 |  |  | 1 | 0.1 |
| Q60 |  |  | 4 | 0.3 |
| Q61 | 1 | 1.0 | 8 | 0.6 |
| Q62 | 5 | 4.9 | 38 | 2.9 |
| Q63 | 1 | 1.0 | 7 | 0.5 |
| Q64 | 2 | 2.0 | 10 | 0.8 |
| Q65 | 10 | 9.8 | 162 | 12.3 |
| Q66 | 12 | 11.8 | 153 | 11.6 |
| Q67 | 3 | 2.9 | 17 | 1.3 |
| Q68 | 1 | 1.0 | 17 | 1.3 |
| Q69 |  |  | 15 | 1.1 |
| Q70 | 2 | 2.0 | 16 | 1.2 |
| Q71 |  |  | 12 | 0.9 |
| Q72 | 1 | 1.0 | 10 | 0.8 |
| Q74 | 1 | 1.0 | 25 | 1.9 |
| Q75 | 2 | 2.0 | 18 | 1.4 |
| Q76 | 1 | 1.0 | 4 | 0.3 |
| Q77 |  |  | 1 | 0.1 |
| Q78 |  |  | 1 | 0.1 |
| Q79 | 1 | 1.0 | 20 | 1.5 |
| Q80 |  |  | 3 | 0.2 |
| Q81 |  |  | 1 | 0.1 |
| Q82 |  |  | 25 | 1.9 |
| Q83 |  |  | 2 | 0.2 |
| Q84 |  |  | 2 | 0.2 |
| Q85 |  |  | 4 | 0.3 |
| Q87 | 4 | 3.9 | 15 | 1.1 |
| Q89 | 5 | 4.9 | 49 | 3.7 |
